# Medical student’s perception of the COVID-19 pandemic effect on their education and well-being: a cross-sectional survey in the United States

**DOI:** 10.1186/s12909-022-03197-x

**Published:** 2022-03-05

**Authors:** Jaideep Chakladar, Anthony Diomino, Wei Tse Li, Joseph C. Tsai, Aswini R. Krishnan, Angela E. Zou, Khush Kharidia, Farhan A. Baig, Sarah Householder, Selena Z. Kuo, Shyam Chandrasekar, Eric Y. Chang, Weg M. Ongkeko

**Affiliations:** 1 Division of Otolaryngology-Head and Neck Surgery, Department Surgery, University of California, La Jolla, San Diego, California USA; 2 Research Service, VA San Diego Healthcare System, San Diego, CA 92161 USA; 3 UC San Diego School of Medicine, University of California, San Diego, CA 92093 USA; 4 Stanford University School of Medicine, Stanford, CA 94305 USA; 5 Harvard Medical School, Harvard University, Boston, MA 02115 USA; 6 Keck School of Medicine, University of Southern California, Los Angeles, CA 90089 USA; 7 University of Missouri-Kansas City School of Medicine, Kansas City, MO 64110 USA; 8 Vagelos College of Physicians and Surgeons, Columbia University, New York, NY 10032 USA; 9 Department of Internal Medicine, Vagelos College of Physicians and Surgeons, Columbia University, New York, NY 10032 USA; 10 Feinberg School of Medicine, Northwestern University, Chicago, IL 60611 USA; 11 Department of Radiology, University of California, San Diego, CA 92093 USA; 12 Radiology Service, VA San Diego Healthcare System, San Diego, CA 92161 USA

**Keywords:** COVID-19, Coronavirus, Pandemic, Medical education, Student wellbeing, United States

## Abstract

**Background:**

The effects of drastic curricular changes necessitated by the COVID-19 pandemic on medical students’ education and wellbeing have remained largely unstudied. Out study aimed to characterize how medical students were affected by the pandemic, specifically how limitations introduced by the pandemic may have affected the quality, delivery, and experience of medical education.

**Methods:**

Three hundred students from 5 U.S. allopathic medical schools were surveyed to determine students’ perceptions about their quality of medical education, professional development, and mental health during the COVID-19 pandemic (October 2020-December 2020).

**Results:**

A large majority of students report that while lecture-based learning has not been significantly affected by the pandemic, small-group and clinical learning have greatly declined in quality. Students also reported higher levels of depression, anxiety, and uncertainty with regards to their futures as physicians.

**Conclusions:**

The COVID-19 pandemic has greatly affected the medical student education and wellbeing. Although medical schools have implemented measures to continue to train medical students as effectively as they can, further strategies must be devised to ensure the well-being of students in the present and for future national emergencies.

**Supplementary Information:**

The online version contains supplementary material available at 10.1186/s12909-022-03197-x.

## Background

The current coronavirus disease 2019 (COVID-19) pandemic has proven to be challenging for people of all walks of life. The medical community has been especially hard hit, considering the hectic and dangerous environments for frontline healthcare providers, the high demand for drug development, and the restrictions on patient care due to safety protocols [1]. Although the effects of the pandemic on medical professionals have been well documented, there has been little investigation into how the pandemic has affected the education and wellbeing of future physicians [2].

The COVID-19 pandemic has widely disrupted education across the United States (U.S.). By Spring 2020, most medical schools in the U.S. halted in-person learning to some degree in accordance with guidelines released by the Association of American Medical Colleges (AAMC) [2]. Medical schools developed virtual lecture and small-group learning formats, halted research projects, and limited in-person clinical experiences. These clinical rotations are often the most pivotal portion of medical training, as students implement preclinical knowledge, directly contribute to patient care, and determine which specialties they will pursue. The inability to work on research projects may severely hinder the amount of research experience certain students may have been hoping to attain, especially those who have taken gap years to pursue research.

The online learning system presents additional variables that may adversely affect medical students’ mental health and general wellbeing. A lack of clinical and research experience may increase anxiety already present in medical students who must soon choose specialties for future residency programs [3]. The inability to gain important experience may decrease medical students’ confidence in their qualifications and make choosing a specialty much more difficult. Medical students who lack comfortable home environments may struggle greatly, as students at other levels have [4]. Extensive use of online communication and learning solutions may prove to be detrimental to student wellbeing, causing increased isolation and conditions such as Zoom fatigue and Burnout syndrome [5–7]. Addition of these factors may only serve to exacerbate metal health problems already common amongst medical students [8].

Lack of in-person learning was projected to have a negative effect on medical education delivery at the start of the pandemic [9], and mandatory social isolation of medical students may negatively affect mental health. Recently, surveys have been conducted to gather opinions from medical students on the rapid shift of medical curriculum to virtual platforms and how limited clinical exposure would impact clinical training and future residency applications [10, 11]. While the most recent studies have characterized the effects of the early stages of the COVID-19 pandemic on medical students and medical education, none have investigated more recent developments, namely the remote clinical experiences and interventions that permit alternative learning experiences. Additionally, there has been little emphasis on the comparison between the effects of the pandemic on students from public and private institutions.

Our present study was designed to gauge the attitudes of medical students after a few months of learning in a COVID-era curriculum. To understand how changes to medical education have affected a diverse pool of medical students, we surveyed students of all class levels from two public and three private medical institutions on their medical education, clinical training, mental wellbeing, and impact on and involvement in the community.

## Methods

### Study design

The current study is a web-based cross-sectional study that utilized an online survey using Qualtrics. This study collected response data from medical students from 5 different institutions in the U.S. between October 11, 2020 and December 3, 2020. We modelled our survey after the survey used by Coffey et al. in their analysis of medical students [12]. Public institutions surveyed included the University of California San Diego School of Medicine (UCSD) and University of Missouri-Kansas City School of Medicine (UKMC). The private institutions surveyed included the University of Southern California’s Keck School of Medicine, Columbia University’s Roy and Diana Vagelos College of Physicians and Surgeons, and Stanford University’s School of Medicine. The survey was designed by three investigators (JC, WTL, WMO) and comprised a total of 26 questions/items, with the number of questions presented to each participant varying based on their responses to previous questions. No identifying information was collected. Participants consented to the use of their responses in this study via the first question of the survey.

### Measures and operationalization

The survey questions consisted of 1 of 3 formats (as determined by Qualtrics): multiple choice, matrix table, and slider. Multiple choice questions were used to collect basic information about participants, including their university, current year in medical school, types of courses they had taken, and types of extracurriculars they were involved in. These questions were placed at the beginning of the survey and were used by Qualtrics to judge which of the subsequent questions would be made available for each individual participant. For example, questions about lab experience during the pandemic would only be presented to a participant if they had indicated in an earlier question that they indeed had lab experience during the pandemic. In order to evaluate differences between students at different levels of medical school, we separated students into 4 groups: first year (MS1s), second year (MS2s), third year (MS3s), and fourth year medical students (MS4s). Matrix table questions were used to gauge the participants’ agreement with certain statements. Agreement was rated on a scale including the following options (in order from least to most agreement): Strongly disagree, Disagree, Somewhat disagree, Neither agree nor disagree, Somewhat agree, Agree, and Strongly agree. Agreement was operationalized using the number of participants that selected each of these options and the total number of times the 3 “agree” statements versus the total number of times the 3 “disagree” statements were selected. Slider questions were used allow participants to indicate the percentage of their courses held virtually, and, if applicable, the number of other individuals (attendings, interns/residents, medical students) they worked with in a clinical setting.

### Survey distribution

Participants were selected via convenience sampling. The survey was distributed to these medical schools using medical student points of contact (POCs) from each of the 5 medical schools. For each school, approval was granted from Offices of Student Affairs to carry out the study after submitting a UCSD IRB waiver, description of the study, and a copy of the proposed survey. POCs were then asked to distribute the survey link to the medical students in their school via email and social media. In order to control for multiple survey entries by a single student, students were required to enter their email address prior to completing the survey. However, participants’ email addresses were not made available by Qualtrics to the authors in order to keep survey data deidentified. The survey remained open from October 11, 2020 to December 3, 2020. Response rate was determined using the total number of concurrently enrolled students at each medical school since data on number of survey recipients was unavailable. The number of concurrently enrolled students at all 5 universities was determined by the Medical School Admission Requirements (MSAR) portal offered by the AAMC. These numbers are as follows: 624 UCSD students, 673 Columbia students, 528 Stanford students, 479 UMKC students, and 815 USC students.

### Statistical analysis

All data was analyzed in either Microsoft Excel or RStudio using base R functions and the tidyverse, dplyr, and ggplot2 packages. Correlations between groups of medical students and their answers to specific questions were analyzed using the Pearson Chi Square statistic or the Fisher's Exact Test (*p*<0.05). The Fisher’s Exact Test was utilized when the number of responses for a specific category was 5 or less.

## Results

### Descriptive statistics

300 responses (~10% response rate) were analyzed from medical students of UC San Diego (22.7%), Stanford (25%), University of Southern California (13%), University of Missouri in Kansas City (12%), Columbia University (26.7%) and others (0.6%). The class level distribution of respondents was 28% MS1s, 33% MS2s, 14.7% MS3s, and 16.7% MS4s. 6.7% students included MD-PhD candidates, Master’s candidates, and gap year students all doing research after their second and third years. Students taking time off for research were combined with their previous level for analysis (i.e., Research year after MS3s were combined with MS3s).

### Efficacy of lecture and small group-based interventions

**Fig. 1:**
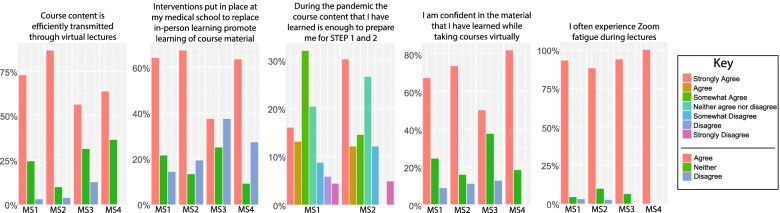
Effects of classroom interventions

The majority of lecture-based classes were held virtually. We asked students how this change affected how they were able to receive and learn course curriculum. Students were largely satisfied with the interventions put in place by their institutions to replace lecture-based learning. 77.2% agreed that course content was properly delivered virtually, and over 60% of students were satisfied with their instructors’ and their institutions’ adaptations to the new format (Fig. 1). However, these changes seemingly cannot completely ease medical student anxiety and replace in-person classes. 91.1% of students reported often experiencing the phenomenon of Zoom fatigue, with 48.6% responding with “Strongly agree” to the prompt (Supplementary Table 1). The United States Medical Licensing Examination (USMLE) is used to provide medical licensing authorities in the U.S. with evidence of qualification for licensure for medical students aspiring to become resident doctors. The USLME consists of three steps, the first two of which are taken during medical school. Step 1 assesses students’ understanding and ability to apply scientific principles to medical practice and is usually taken by the end of the scholastic year by MS2s. Step 2CK, or Step 2, assesses students’ ability to apply medical knowledge in a clinical setting. It is usually taken by MS3s after they have finished their core rotations. Only 50% of respondents report that the course material they are learning will prepare them for Step 1 and Step 2 (Fig. 1). 36.8% of students agreed that the amount they are learning is the same amount of material learned by students of previous years (Supplementary Table 1). Together, this data suggests that, despite the accessibility of information and faculty by medical students, there are significant challenges presented by the pandemic that make students feel less confident in the material that they have learned.

Small-group learning classes, which revolve around in-person group discussion, also shifted to remote formats. Most students reported that 95% or more of their small group classes, including Problem-Based Learning (PBL) and, Principles of Medicine (POM), went virtual as a result of the pandemic. Students were given separate prompts asking them to evaluate whether or not virtual versions of their small group classes were sufficient substitutions for their in-person versions. Out of the three types of small group classes, virtual POM classes were viewed most unfavorably, as 73.2% of respondents disagreed that virtual POM is a sufficient substitute for in-person POM. Virtual PBL classes and other small group classes were viewed more favorably, as 54.8% and 45.6% of respondents thought virtual versions of those classes were sufficient substitutions for in-person versions (Supplementary Table 1).

### Extracurricular community service and learning opportunities

**Fig. 2:**
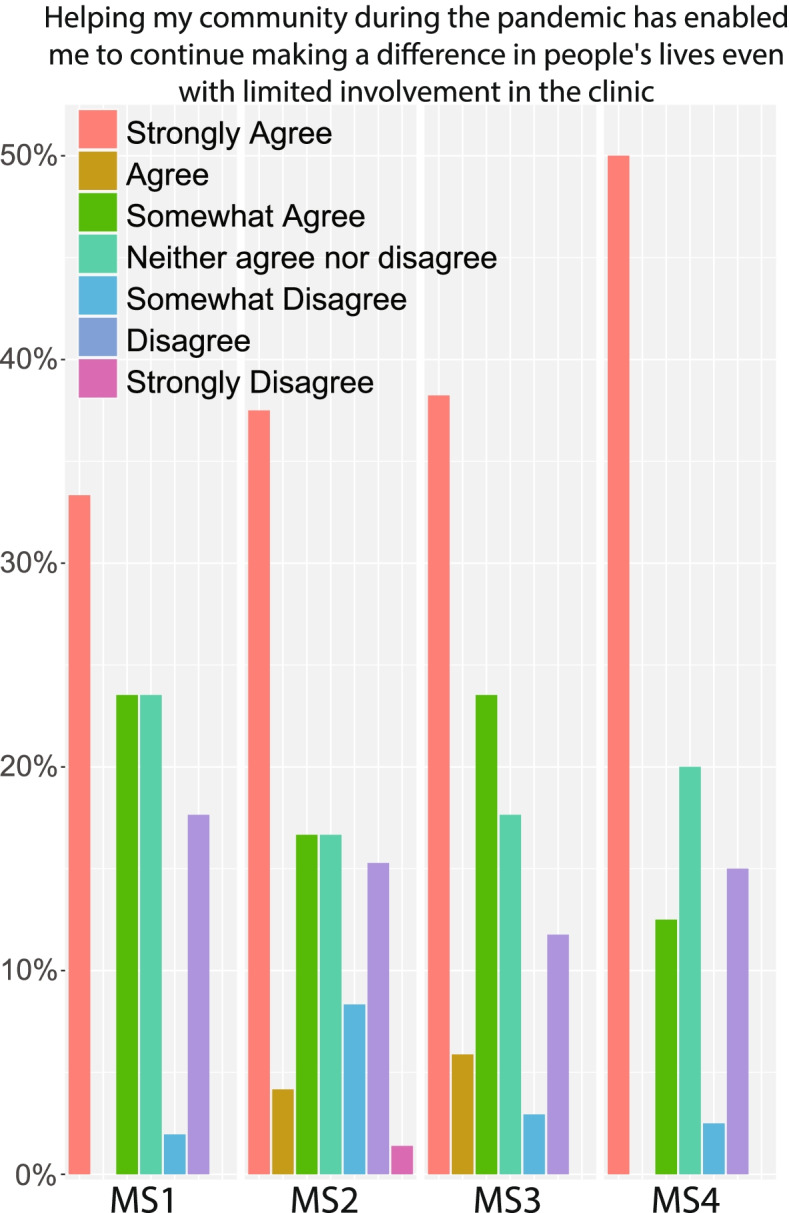
Effects of extracurricular community service on students’ mindset

Due to restrictions imposed by the pandemic, medical students may feel disconnected from traditional extracurricular activities involving professional development, social engagement, or community service. We asked students whether they had looked for other outlets in order to address this disconnect. Aside from course material, 73.7% of students reported initiating or participating in opportunities that provided other ways to learn. Many students cited engaging in ‘technical support’, ‘creating support groups’, and ‘creating study groups’. 33.6% of students also responded that they were involved in teaching other medical or Science, technology, engineering, and mathematics (STEM) students at some point since the pandemic started. Of the students that engaged in such activities, a large majority believed that these activities allowed them to continue to make a difference (Fig. 2). This may indicate the importance of such extracurriculars to the well-being of medical students while other avenues of service are hindered by the pandemic.

### Virtualization and limitation of clinical rotations

**Fig. 3:**
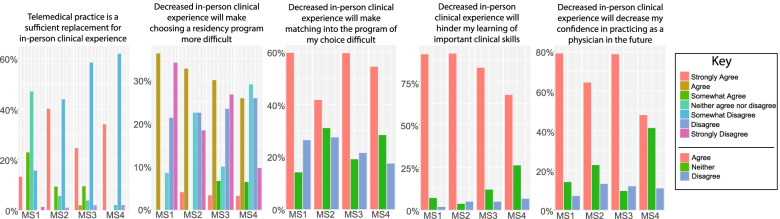
Efficacy of abbreviated clinical experience and its effects on feelings of preparedness

The majority of students in clinical rotations either had to perform them virtually or in a heavily abbreviated manner (Supplementary Table 1). Among preclinical students, only 30.7% agreed that the course content they were learning was sufficient to prepare them for clinical rotations, and 44% of clinical-level students agreed that the skills they were learning would be enough to prepare them for residency. 54.7% of clinical-level students agreed that there was a decrease in the amount of clinical training they were receiving. When asked about their opinions on how clinical training might affect their ability to choose a specialty, only 37.4% agreed that the skills and content learned while on clinical rotations would help them discern their desired specialty (Fig. 3).

In terms of medical team composition while in the clinic, most students cited working with 1 attending, 1 resident, 1 other medical student in their ward teams. This is a decrease from the average number of colleagues and supervisors that medical students are able to work with during rotations with larger teams, such as those in general surgery where students work with multiple medical students and residents.

### Research projects

58.5% of students reported that the pandemic affected their research projects negatively. 41.9% of respondents also reported that, because of the pandemic, they had not gained the skills they expected to while doing research, and 61.9% stated that their research was slowed. There were no significant differences in responses between students pursuing part-time research and students pursuing full-time research (gap year, PhD, MS).

### Mental wellbeing and anxiety for the future

**Fig. 4:**
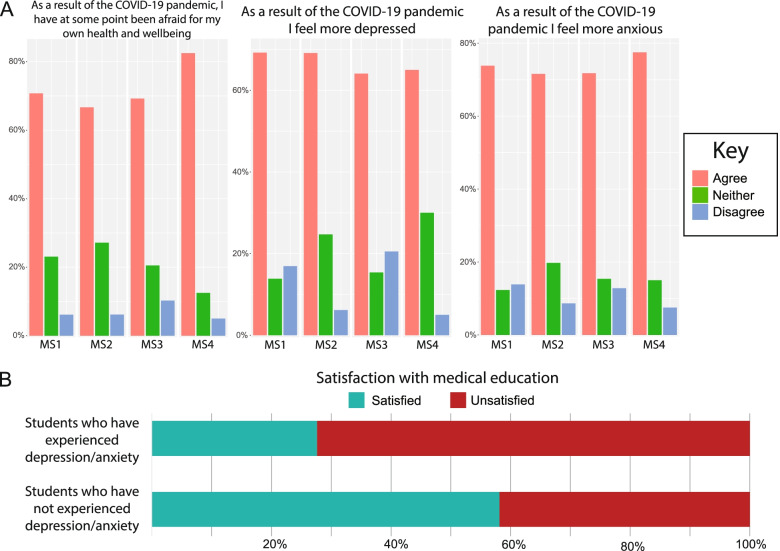
The effect of the pandemic on medical students’ mental health. **A**. Agreement with statements concerning wellbeing, depression, and anxiety. **B**. Correlation between satisfaction with medical education (relative to perception of previous medical students)

Students were asked about how the pandemic affected their mental health as it related to depression, anxiety, and sources of support. 88.4% of all prompted students report the pandemic causing an increased sense of isolation from peers. Many students also report the pandemic being a factor in increased feelings of depression (67.5%) and anxiety (73%). Students were not asked to clarify whether this depression was clinically diagnosed or not. When asked if, during the pandemic, they had ever been afraid for their own health and wellbeing, 71% of students indicated agreement. In addition, a number of students reported strong agreement with the pandemic influencing feelings of isolation, depression, and anxiety (37.7%, 16%, 20.8% respectively) (Fig. 4A). Students who reported experiencing feelings of depression or anxiety as a result of the pandemic were also less satisfied with their medical education than students who had not experienced such feelings (Fig. 4B).

**Fig. 5:**
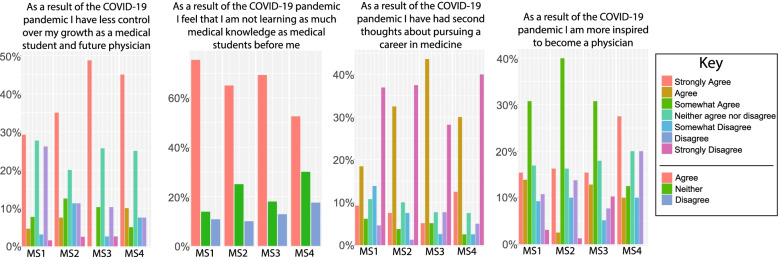
Anxiety for the future and commitment to medicine

We administered a group of questions to gauge how the pandemic has affected students’ thoughts and beliefs about their futures in medicine. 81% of students believed that the pandemic has lessened their control over their growth as students and as future physicians. 70.9% of students felt that their medical knowledge is behind that of medical students that came before them (Fig. 5). Interestingly, students who reported experiencing feelings of depression or anxiety were more likely to be dissatisfied with their medical education in comparison to students who had not experienced such feelings (Fig. 4B). Although 52.9% of applicants thought that the pandemic further inspired them to become physicians, an alarming 31.2% of students expressed second thoughts about pursuing a career in medicine (Fig. 5). Interestingly, MS4s were seemingly the most confident in their abilities, education, and future trajectories. This may be due to the fact that MS4 students have already gained confidence after at least 3 years of medical school, mostly were in elective rotations, and have already decided on their field of specialty based on their previous experience in core rotations.

### Differences between the public versus private university experience

**Fig. 6:**
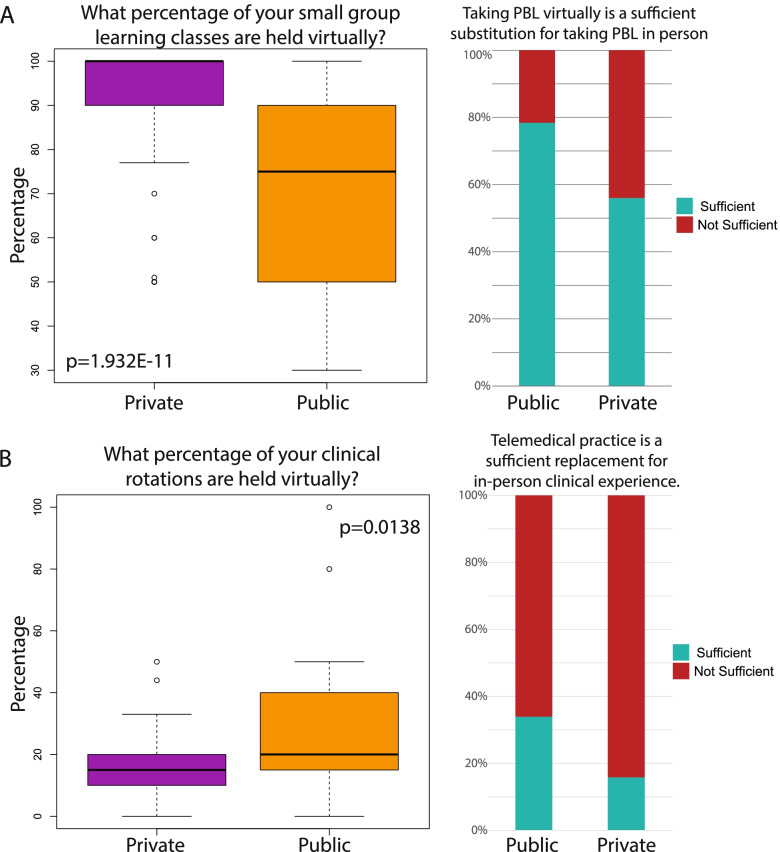
The effects of the pandemic on public versus private institutions. Significant differences were between perceptions of **A**. virtual small group learning and **B**. virtual clinical practice

Differences in responses between students from the two public and three private medical schools were analyzed using the Kruskal Wallis test (*p*<0.05). Public school students reported having fewer virtual small group learning classes and 70.6% of students who had taken virtual PBL courses did not believe that they were a sufficient substitute for in-person classes (Fig. 6A). In general, private school students reported being more satisfied with the replacement of in-person clinical experience with telemedical practice. In private schools, a lower percentage of clinical rotations were held virtually compared to rotations in public schools. More than public school students, private school students reported that their experience with telemedical rotations was a sufficient replacement to in-person clinical rotations (Fig. 6B). Other than these findings, there were no significant differences between public school and private school student responses. However, it is important to note that the sample size for this analysis is very low (2 private schools vs 3 public schools). Our data may therefore not be generalizable to all institutions.

## Discussion

Because the COVID-19 pandemic has so deeply affected the US education system in general, students and faculty must adjust in order to facilitate a transfer of knowledge that would maximally approach what students would experience pre-pandemic. This is especially true for medical students, as the skills learned in medical school form the foundation of much more complex skills to be used by these future physicians. Prior to the pandemic, the mental health epidemic that plagues medical students was well-characterized [13]. The exacerbation of mental health problems and roadblocks to attaining medical knowledge may deeply affect the generation of future physicians training through the pandemic.

Across the world, medical students have reported dissatisfaction with online medical school education. In the U.S., a survey of 1,389 medical students revealed that online education significantly decreased students’ overall wellness [14]. Two separate studies from Jordan reported that 26.8% and 25% of medical students, respectively, were not satisfied with their online education [15, 16]. In a survey of an Indian medical school, over half of the respondents prefer physical classes over online classes [17]. In a study in Saudi Arabia, only 25% of students prefer online only classes over integrated or in-person only classes [18] . In our study, a similar dissatisfaction with online medical education and negative mental health impact were noted. Similar to the studies referenced above, we found that 70% of students surveyed believed that their quality of education declined (they do not learn as much as students before them). Despite the numerous studies done on the subject, our study is the first, to the best of our knowledge, to document the potential causes of student dissatisfaction, including mental health obstacles, decline in quality of group discussions, etc.

We found that the transition to online lecture-based classes has not severely affected students’ self-reported learning. Students largely feel that virtual lecture-based learning still allows for material to be effectively communicated and absorbed. Although students do experience a significant amount of Zoom fatigue, the majority of them believe that the material they learn is sufficient for the USMLE. A significant number of students, however, believed that the substitutions for small group learning classes were insufficient. As these classes are important for developing critical thinking skills and application of lecture-based knowledge, the dissatisfaction with virtual small group learning courses may contribute to medical students’ overall apprehension regarding the adequacy of amount of material that they are learning.

Although clinical rotations were not completely halted, the majority operated virtually or under strict restrictions. Most students reported working with smaller teams compared to the size of clinical teams pre-pandemic. The utility of clinical rotations hinges on interactions with patients and with various members of the medical team. Given that both types of interactions were significantly limited, it is not surprising that a majority of students found their clinical experience to be lacking. With students feeling less prepared to apply to and enter residency programs, it may be important to find ways in which remote or abbreviated clinical rotations may be improved.

A great majority of students presumably enter medical school to fulfill a desire to help others by being healers, but this pandemic may have made some of these students feel less able to serve others. We found that students who found other ways to benefits others in their community believed their experience to be very rewarding, as they were able to further develop their interpersonal and emotional maturity outside of the clinic. Utilizing these types of community service opportunities may help to address problems that medical students have when unable to do the things that drew them to a career in medicine.

The overall rate of feelings of depression and anxiety during the COVID-19 pandemic were about two times higher than the average prevalence of depression or depressive symptoms in medical students, according to previous meta-analysis [19]. This alarming increase may be attributed to multiple causes. It has been reported that medical students who feel anxious or depressed are likely to have these feelings increase in severity and frequency once they enter the workforce [20]. However, those that develop coping mechanisms are much less likely to experience such symptoms. These coping mechanisms are usually interaction with friends or family, leisure and exercise, and positive outlooks [21]. The pandemic, by all accounts, has negatively impacted these coping mechanisms. Students who are not receiving the education that they were expecting may also feel a degree of helplessness, a hypothesis supported by the majority of students we found to feel less certain and in control of their education and futures. Despite these conclusions, there are several limitations to our study. During data collection, we were unable to obtain data on how many students received our survey, as it was distributed through social media. We therefore could not calculate the most accurate response rate as we utilized the estimated total class size of each medical school as a proxy for our lacking data. In terms of representativeness, we attempted to obtain responses from both public and private schools and from multiple locations around the US. However, due to more than half of survey responses coming from students in universities at the US coasts and in larger metropolitan cities, it may be difficult to generalize our findings to students studying at medical schools located in other areas of the US. As this study was not a prospective study, there is potential for recall bias in survey responses. We attempted to limit this bias by gaging overall feelings towards the pandemic and medical education in our questions and recognizing whether there was a correlation of those feels to more positive or negative responses. However, our data indicated that there was no such consistent correlation across all attitude-related questions. We therefore believe that the effect of recall bias in our study was limited. Previous studies of depression in the student population mainly focus on clinically diagnosed depression, rather than just depressive symptoms. It is most likely that the majority of medical students who reported depressive or anxious episodes in our study are not clinically depressed. Regardless, it is still important to monitor depressive symptoms, considering the common trajectory that medical students take towards depression as they enter the workforce. Finally, our survey also did not explore the availability and use of COVID-related mental services to medical students. Many institutions such as UC San Diego and Stanford saw an increase in mental health appointments overall. These increases reflected a national rise in mental health disability [22]. Future surveys can investigate the effectiveness of mental health services in dealing with mental health cases among medical students, amidst increased pressure related to school, research, and clinical training.

## Conclusion

In all, our study indicates that the pandemic has significantly impacted the lives of medical students. Many students were able to develop some coping mechanisms and did not waver from their path towards becoming physicians. However, the alarming number of students with second thoughts about their choice of profession, depression, anxiety about the future, and perceived lack of training indicate that medical students should be provided further resources to address these problems.

## Supplementary Information

**Additional file 1.**

**Additional file 2.**

## Data Availability

The datasets used and analysed during the current study are available from the corresponding author on reasonable request.
