# Supplementary material for: Medical student’s perception of the COVID-19 pandemic effect on their education and well-being: a cross-sectional survey in the United States
Source: BMC Med Educ. 2022 Mar 5;22:149. doi: 10.1186/s12909-022-03197-x (PMC8897763; doi:10.1186/s12909-022-03197-x)
Supplement: Supplementary file 2 — Additional file 2. [file 12909_2022_3197_MOESM2_ESM.doc]

Qualtrics Survey Instrument

Start of Block: General Questions

1 Check the box below if you consent to have your responses used for further study

- Yes (1)

2 What medical school do you currently attend?

- UC San Diego (1)
- Stanford (2)
- Harvard (3)
- UMKC (4)
- USC (5)
- Northwestern (6)
- Columbia (8)
- Other (7) ________________________________________________

3 What is your current year?

- MS1 (1)
- MS2 (2)
- Research year after MS2 (5)
- MS3 (3)
- Research year after MS3 (6)
- MS4 (4)

End of Block: General Questions

Start of Block: Clinical Experience

4 How have you interacted with patients during the pandemic? Check all that apply.

- Virtually via telemedical services (1)
- In-person (2)
- No patient interaction (3)

Display This Question:

If How have you interacted with patients during the pandemic? Check all that apply. = Virtually via telemedical services

5 Rate your agreement with the following statement: Telemedical practice is a sufficient replacement for in-person clinical experience.

- Strongly disagree (1)
- Disagree (2)
- Somewhat disagree (3)
- Neither agree nor disagree (4)
- Somewhat agree (5)
- Agree (6)
- Strongly agree (7)

Display This Question:

If How have you interacted with patients during the pandemic? Check all that apply. = In-person

Or How have you interacted with patients during the pandemic? Check all that apply. = Virtually via telemedical services

6 While evaluating patients in an in-person clinical setting during the pandemic, how many others (medical students, attendings, residents, etc.) work with you?

|  | 0 | 1 | 2 | 3 | 4 | 5 | 6 | 7 | 8 | 9 | 10 | 11 | 12 | 13 | 14 | 15 | 16 | 17 | 18 | 19 | 20 |
| --- | --- | --- | --- | --- | --- | --- | --- | --- | --- | --- | --- | --- | --- | --- | --- | --- | --- | --- | --- | --- | --- |

| Attending () | 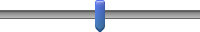 |
| --- | --- |
| Interns/Residents () | 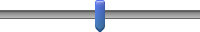 |
| Medical Students () | 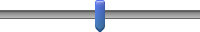 |
| Others () | 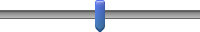 |

Display This Question:

If How have you interacted with patients during the pandemic? Check all that apply. = Virtually via telemedical services

Or How have you interacted with patients during the pandemic? Check all that apply. = In-person

7 Rate your agreement with the following statements: Decreased in-person clinical experience as a result of the pandemic will:

|  | Strongly disagree (6) | Disagree (7) | Somewhat disagree (8) | Neither agree nor disagree (9) | Somewhat agree (10) | Agree (11) | Strongly agree (12) |
| --- | --- | --- | --- | --- | --- | --- | --- |
| Make choosing a residency program more difficult (1) |  |  |  |  |  |  |  |
| Make matching into the program of my choice difficult (2) |  |  |  |  |  |  |  |
| Hinder my learning of important clinical skills (3) |  |  |  |  |  |  |  |
| Decrease my confidence in practicing as a physician after medical school (4) |  |  |  |  |  |  |  |

End of Block: Clinical Experience

Start of Block: Effects on Courses and Rotations

8 Which of the following programs or courses have you participated in during the pandemic?

- Lecture-based courses (442)
- Small group learning courses (ex: problem based learning) (443)
- Lab-based courses (ex: Anatomy, Histology, Ultrasound) (444)
- Ambulatory Care (445)
- Pre-clinical electives (446)
- Core rotations (447)
- Elective rotations (448)
- Research rotations/independent research (449)
- Other (450) ________________________________________________

Display This Question:

If Which of the following programs or courses have you participated in during the pandemic? = Lecture-based courses

9 What percentage of your lecture-based classes are held virtually?

|  | 0 | 10 | 20 | 30 | 40 | 50 | 60 | 70 | 80 | 90 | 100 |
| --- | --- | --- | --- | --- | --- | --- | --- | --- | --- | --- | --- |

| Percentage () | 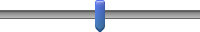 |
| --- | --- |

Display This Question:

If Which of the following programs or courses have you participated in during the pandemic? = Small group learning courses (ex: problem based learning)

10 What percentage of your small group learning classes are held virtually?

|  | 0 | 10 | 20 | 30 | 40 | 50 | 60 | 70 | 80 | 90 | 100 |
| --- | --- | --- | --- | --- | --- | --- | --- | --- | --- | --- | --- |

| Percentage () | 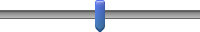 |
| --- | --- |

Display This Question:

If Which of the following programs or courses have you participated in during the pandemic? = Lab-based courses (ex: Anatomy, Histology, Ultrasound)

11 What percentage of your laboratory classes are held virtually?

|  | 0 | 10 | 20 | 30 | 40 | 50 | 60 | 70 | 80 | 90 | 100 |
| --- | --- | --- | --- | --- | --- | --- | --- | --- | --- | --- | --- |

| Percentage () | 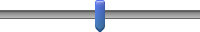 |
| --- | --- |

Display This Question:

If Which of the following programs or courses have you participated in during the pandemic? = Core rotations

Or Which of the following programs or courses have you participated in during the pandemic? = Elective rotations

12 What percentage of your clinical rotations are held virtually?

|  | 0 | 10 | 20 | 30 | 40 | 50 | 60 | 70 | 80 | 90 | 100 |
| --- | --- | --- | --- | --- | --- | --- | --- | --- | --- | --- | --- |

| Percentage () | 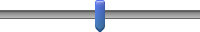 |
| --- | --- |

Display This Question:

If Which of the following programs or courses have you participated in during the pandemic? = Small group learning courses (ex: problem based learning)

13 Indicate which courses you have taken during the pandemic

- Problem-Based Learning (PBL) or similar course (1)
- Practice of Medicine (POM) or similar course (2)
- Other Small group learning courses (3) ________________________________________________
- None of the above (4)

Display This Question:

If Indicate which courses you have taken during the pandemic = Problem-Based Learning (PBL) or similar course

14 Rate your agreement with the following statements: Taking PBL virtually is a sufficient substitution for taking PBL in-person.

- Strongly disagree (1)
- Disagree (2)
- Somewhat disagree (3)
- Neither agree nor disagree (4)
- Somewhat agree (5)
- Agree (6)
- Strongly agree (7)

Display This Question:

If Indicate which courses you have taken during the pandemic = Practice of Medicine (POM) or similar course

15 Rate your agreement with the following statements: Taking POM virtually is a sufficient substitution for taking POM in-person.

- Strongly disagree (1)
- Disagree (2)
- Somewhat disagree (3)
- Neither agree nor disagree (4)
- Somewhat agree (5)
- Agree (6)
- Strongly agree (7)

Display This Question:

If Indicate which courses you have taken during the pandemic = Other Small group learning courses

16 Rate your agreement with the following statements: Taking small group learning courses virtually is a sufficient substitution for taking them in-person.

- Strongly disagree (1)
- Disagree (2)
- Somewhat disagree (3)
- Neither agree nor disagree (4)
- Somewhat agree (5)
- Agree (6)
- Strongly agree (7)

Display This Question:

If What percentage of your lecture-based classes are held virtually? [ Percentage ] > 0

Or What is your current year? = MS1

Or What is your current year? = MS2

Or What is your current year? = Research year after MS2

17 Rate your agreement with the following statements as they relate to virtual lectures

|  | Strongly disagree (1) | Disagree (2) | Somewhat disagree (3) | Neither agree nor disagree (4) | Somewhat agree (5) | Agree (6) | Strongly agree (7) |
| --- | --- | --- | --- | --- | --- | --- | --- |
| I often experience Zoom fatigue during lectures. (1) |  |  |  |  |  |  |  |
| Course content is efficiently transmitted through virtual lectures. (2) |  |  |  |  |  |  |  |
| Faculty members are effectively able to use online learning tools. (3) |  |  |  |  |  |  |  |
| I am confident in the material that I have learned while taking courses virtually. (4) |  |  |  |  |  |  |  |
| Interventions put in place at my medical school to replace in-person learning promote learning of course material. (5) |  |  |  |  |  |  |  |

Display This Question:

If What is your current year? = MS1

Or What is your current year? = MS2

Or What is your current year? = Research year after MS2

18 Rate your agreement with the following statements: During the pandemic, the course content that I have learned is

|  | Strongly disagree (1) | Disagree (2) | Somewhat disagree (3) | Neither agree nor disagree (4) | Somewhat agree (5) | Agree (6) | Strongly agree (7) |
| --- | --- | --- | --- | --- | --- | --- | --- |
| enough to prepare me for STEP 1 and 2. (1) |  |  |  |  |  |  |  |
| equal to the amount learned by medical students before me. (2) |  |  |  |  |  |  |  |
| enough to prepare me for clinical rotations. (3) |  |  |  |  |  |  |  |

Display This Question:

If What is your current year? = MS3

Or What is your current year? = MS4

Or What is your current year? = Research year after MS2

Or What is your current year? = Research year after MS3

Or Which of the following programs or courses have you participated in during the pandemic? = Core rotations

Or Which of the following programs or courses have you participated in during the pandemic? = Elective rotations

Or Which of the following programs or courses have you participated in during the pandemic? = Research rotations/independent research

19 Rate your agreement with the following statements: During the pandemic, the skills and content that I have learned while on clinical rotations

|  | Strongly disagree (1) | Disagree (2) | Somewhat disagree (3) | Neither agree nor disagree (4) | Somewhat agree (5) | Agree (6) | Strongly agree (7) |
| --- | --- | --- | --- | --- | --- | --- | --- |
| is enough to prepare me for residency. (1) |  |  |  |  |  |  |  |
| is equal to the amount learned by medical students before me. (2) |  |  |  |  |  |  |  |
| helps me discern what specialty I would like to go into. (3) |  |  |  |  |  |  |  |

Display This Question:

If What is your current year? = Research year after MS2

Or What is your current year? = Research year after MS3

Or Which of the following programs or courses have you participated in during the pandemic? = Research rotations/independent research

Q27 Rate your agreement with the following statements: Because of the pandemic,

|  | Strongly Disagree (1) | Disagree (2) | Somewhat disagree (3) | Neither agree nor disagree (4) | Somewhat agree (5) | Agree (6) | Strongly agree (7) |
| --- | --- | --- | --- | --- | --- | --- | --- |
| my research has been negatively impacted. (1) |  |  |  |  |  |  |  |
| I have not been able to gain the skills that I expected to gain when going into research. (2) |  |  |  |  |  |  |  |
| my research projects have been paused or severely slowed. (3) |  |  |  |  |  |  |  |
| my research-related work is less applicable to the medical field. (4) |  |  |  |  |  |  |  |

End of Block: Effects on Courses and Rotations

Start of Block: General Health

Q25 As a result of the pandemic, have you initiated or participated in opportunities that provide alternative ways to

|  | Yes (1) | No (2) |
| --- | --- | --- |
| learn? (1) |  |  |
| teach other medical and STEM students? (2) |  |  |
| volunteer in your community? (3) |  |  |
| care for patients? (4) |  |  |

Q28 How have you helped faculty or fellow students with problems brought on by the pandemic?

- Technical support (1)
- Tutoring (2)
- Creating study groups (3)
- Creating support groups (4)
- None of the above (5)

Display This Question:

If As a result of the pandemic, have you initiated or participated in opportunities that provide alt... = volunteer in your community? [ Yes ]

Or As a result of the pandemic, have you initiated or participated in opportunities that provide alt... = teach other medical and STEM students? [ Yes ]

Or As a result of the pandemic, have you initiated or participated in opportunities that provide alt... = care for patients? [ Yes ]

Or How have you helped faculty or fellow students with problems brought on by the pandemic? != None of the above

Q29 Rate your agreement with the following statements: Helping my community during the pandemic has enabled me to

|  | Strongly Disagree (1) | Disagree (2) | Somewhat disagree (3) | Neither agree nor disagree (4) | Somewhat agree (5) | Agree (6) | Strongly agree (7) |
| --- | --- | --- | --- | --- | --- | --- | --- |
| feel more personally fulfilled. (1) |  |  |  |  |  |  |  |
| gain an increased sense of empathy for others. (2) |  |  |  |  |  |  |  |
| feel more compassionate towards members of my community. (3) |  |  |  |  |  |  |  |
| continue making a difference in people's lives even with limited involvement in the clinic. (4) |  |  |  |  |  |  |  |

20 Rate your agreement with the following statements: During the COVID-19 pandemic,

|  | Strongly disagree (1) | Disagree (2) | Somewhat disagree (3) | Neither agree nor disagree (4) | Somewhat agree (5) | Agree (6) | Strongly agree (7) |
| --- | --- | --- | --- | --- | --- | --- | --- |
| I feel connected to my medical school. (1) |  |  |  |  |  |  |  |
| I can work together with other students and faculty during courses and/or rotations. (2) |  |  |  |  |  |  |  |
| I can communicate with faculty members. (3) |  |  |  |  |  |  |  |
| I can communicate with other medical students. (4) |  |  |  |  |  |  |  |

21 Rate your agreement with the following statements: As a result of the COVID-19 pandemic,

|  | Strongly disagree (1) | Disagree (2) | Somewhat disagree (3) | Neither agree nor disagree (4) | Somewhat agree (5) | Agree (6) | Strongly agree (7) |
| --- | --- | --- | --- | --- | --- | --- | --- |
| I have, at some point, been afraid for my own health and wellbeing. (1) |  |  |  |  |  |  |  |
| I feel more depressed. (2) |  |  |  |  |  |  |  |
| I feel more anxious. (3) |  |  |  |  |  |  |  |
| my support system(s) has become less effective. (4) |  |  |  |  |  |  |  |
| I feel more isolated from my peers. (5) |  |  |  |  |  |  |  |

Q32 Rate your agreement with the following statements: As a result of the COVID-19 pandemic,

|  | Strongly disagree (8) | Disgree (9) | Somewhat disagree (10) | Neither agree nor disagree (11) | Somewhat agree (12) | Agree (13) | Strongly agree (14) |
| --- | --- | --- | --- | --- | --- | --- | --- |
| I have less control over my growth as a medical student and future physician. (1) |  |  |  |  |  |  |  |
| I feel that I am not learning as much medical knowledge as medical students before me. (2) |  |  |  |  |  |  |  |
| I have had second thoughts about pursuing a career in medicine. (3) |  |  |  |  |  |  |  |
| I am more inspired to become a physician. (4) |  |  |  |  |  |  |  |

End of Block: General Health
